# Skin of color representation in dermoscopy studies distinguishing benign from malignant skin lesions: A scoping review

**DOI:** 10.1016/j.jdin.2023.08.018

**Published:** 2023-09-09

**Authors:** Lauren M. Fahmy, Ioanna Maria Karantza, Celine M. Schreidah, Brigit A. Lapolla, Larisa J. Geskin

**Affiliations:** a Columbia University Vagelos College of Physicians and Surgeons, New York, New York; b Department of Dermatology, Columbia University Irving Medical Center, New York, New York

**Keywords:** acral lentiginous melanoma, basal cell carcinoma, dermoscopy, health disparities, melanoma, racial and ethnic representation, skin cancer, skin of color, squamous cell carcinoma

*To the Editor:* Dermoscopy is a promising tool that can enhance the diagnosis of cutaneous malignancies.^1^ To ensure that the benefits of this instrument are equitable, it is important to include diverse patient populations in research used to develop and validate diagnostic criteria. Lack of diversity may result in dermoscopic criteria that either under- or over-diagnose malignancy in under-represented populations. This study evaluated participant diversity in studies examining dermoscopic features distinguishing benign from malignant skin lesions.

PubMed was searched for peer-reviewed articles examining dermoscopic features of melanoma, squamous cell carcinoma, and basal cell carcinoma published from inception to January 3, 2023. Search terms can be found in Supplemental Text 1, available via Mendeley at https://doi.org/10.17632/3sxzh464tj.2. In total, 2470 articles were identified; titles and abstracts were screened, and 472 full-text articles were independently reviewed by 2 investigators (LMF, IMK) with conflicts resolved by discussion. Included studies were required to demonstrate histologically-proven malignant lesions, including >5 patients, and assess the ability of at least 1 dermoscopic feature to differentiate benign from malignant skin lesions. Studies that were unavailable in English, utilized computer-aided diagnosis, related to conjunctival melanoma, or review articles were excluded. Purely descriptive studies (ie, those lacking inferential statistics) were excluded since these studies are not rigorous enough to draw generalizable conclusions. A total of 159 studies met inclusion criteria.

Among the 159 studies, only 8 (5.0%) reported subject race and/or ethnicity, with 7/8 studies including White patients. Only 1 study explicitly included Black, Native American, or Pacific Islander patients, 2 studies included Hispanic patients, and 4 studies included Asian patients. Phototype was reported in 21/159 (13.2%) studies, with most including participants with phototypes I-III, fewer including phototypes IV-V, and none including phototype VI. Nationality was disclosed in 12/159 (7.5%) studies (Table I).

**Table I:** Representation of diverse populations in dermoscopy studies distinguishing melanoma, BCC, and/or SCC from benign lesions

Characteristics	No. of studies		
	All included studies	Studies including acral lentiginous melanoma∗	Top 20 most highly cited publications
Total	159	19	20
Report race and/or ethnicity (%)	8 (5.0)	3 (15.7)	0 (0)
Explicitly include patients with the following races and ethnicities†			
White	7	3	0
Black	1	1	0
Hispanic	2	1	0
Asian	4	2	0
Native American	1	1	0
Pacific Islander	1	1	0
Report skin phototype (%)	21 (13.2)	4 (21.1)	1 (5.0)
Explicitly include patients with the following skin phototypes†			
I	14	2	1
II	17	2	1
III	19	3	1
IV	9	3	1
V	3	2	0
VI	0	0	0
Report nationality (%)	12 (7.5)	5 (26.3)	1 (5.0)
Explicitly include patients with the following nationalities†			
Brazilian	1	0	0
Iranian	1	1	0
Italian	2	1	0
Japanese	3	1	1
Korean	2	1	0
Polish	1	0	0
Thai	1	1	0
Turkish	1	0	0

*BCC*, Basal cell carcinoma; *SCC*, squamous cell carcinoma.

∗ Includes acral lentiginous melanoma, acral lentiginous melanoma in situ, nail unit/apparatus melanoma, nail unit/apparatus melanoma in situ.

† Studies with patients from multiple categories were included more than once.

Because acral lentiginous melanoma (ALM) comprises a larger proportion of skin malignancies in skin of color (SOC) patients,^2^ a subset of 19/159 studies including ALM was examined. Among 3/19 (15.8%) studies reporting race and ethnicity and 4/19 (21.1%) reporting phototype, most patients were White or light-skinned (phototypes I-III) (Table I).

We examined the top 20 most highly cited publications among the included studies, as these are likely to have the largest impact on clinical practice. None reported race or ethnicity, 1 reported phototype (with most participants in phototypes I/II), and 1 reported patient nationality (Table I).

In conclusion, most included dermoscopy studies did not report patient race, ethnicity, or phototype, making it difficult to assess the representation of diverse skin tones and/or racial and ethnic backgrounds. Few studies included non-White and non-Asian racial and ethnic groups and darker skin phototypes, indicating potential underrepresentation. Such under-representation may inadvertently harm SOC patients, given that dermoscopic patterns may vary across different skin tones.^3^^,^^4^ Alternative criteria with better diagnostic accuracy in darker skin tones may exist. Future studies should address this knowledge gap and consider the use of more inclusive skin pigmentation scales.^5^ Our findings underscore the need for increased reporting of patient race, ethnicity, and skin tone in dermoscopy studies.

## Conflicts of interest

LJG has served as an investigator for and/or received research support from Helsinn Group, J&J, Mallinckrodt, Kyowa Kirin, Soligenix, Innate, Merck, BMS, and Stratpharma; on the speakers’ bureau for Helsinn Group and J&J; and on the scientific advisory board for Helsinn Group, J&J, Mallinckrodt, Sanofi, Regeneron, and Kyowa Kirin. LMF, IMK, CMS, and BAL have no conflicts of interest to declare.
